# The Phoenix Fallacy: Reframing the Irreversible Transformations Following Workplace Burnout in Autistic Adults

**DOI:** 10.1089/aut.2024.0292

**Published:** 2025-01-27

**Authors:** Stéphane de Leymarie

**Affiliations:** Faculty of Health and Life Sciences, Department of Psychology, University of Exeter, Exeter, United Kingdom.

April 13, 2022, 7:32 PM. I am looking at my right hand, which refuses to move as if it is screwed to the computer mouse. My vision is blurred, my field of vision narrowed, my body frozen. Nothing in me seems to respond anymore. Out of the deafening silence of the laboratory where I am pursuing my PhD, a blank void of several hours swallowed me whole. My next memory is of sitting at home at my work desk upstairs. For several hours, my body cries all its tears, and there is nothing I can do but watch the terrible and painful scene of my collapse, waiting for my body to finish vomiting these endless waves of tears and nerve convulsions—another black hole.

The following day, I am staring at my computer screen, hearing my general practitioner pronounce words that resonate in my cells like a terrifying relief: “You are in severe burnout. You are stopping work. Right now.” Immediately, two conflicting sensations mingle: an immense, almost salvational relief and the feeling that the ground is falling from under my feet.

 Three years earlier, at the age of 35, I was diagnosed with Level 1 Autism Spectrum Disorder (ASD), central hyperacusis, dyslexia, and dyscalculia; precious keys of understanding, although late, whose full implications I was only beginning to grasp in my daily life when I started my PhD in April 2021. It was a moment of triumph: I had successfully changed my career path and returned to university. I was finally on the cusp of achieving my dream of becoming a brain and mind researcher. I was still grappling with deeply ingrained compensatory reflexes, but I was managing well and felt hopeful. At that time, I was a thousand miles away from imagining that almost exactly 12 months later, I would find myself in that cold, silent laboratory—exhausted, annihilated, powerless—succumbing to this wild and ravaging yet eerily silent fire of burnout.

At first glance, the open space we shared with other research institutes appeared welcoming, bathed in warm light streaming through the large bay windows that lined the floor. The view was splendid; the facilities were new. There was a kitchen, a dining area, and a lounge—all the comfort of modern offices. When they first showed me my workspace, I couldn’t help but notice a slight discomfort within me: How am I going to manage in this space? In front of me, to my right, and just a meter behind, were colleagues and unfamiliar individuals from other organizations. Daily life in this space quickly became problematic. The light from outside blinded me, but most of the floor wanted to keep the blinds up. The clicking of fingers on computer keyboards pierced my body like thousands of tiny needles. The vibrating neon light above me was making me nauseous. The smell of food heated in the microwave filled the workspace, seeping into my pores and paralyzing my mind. The regular swallowing of my colleague on the right twisted my insides. The constant chatter further thickened the sensory cacophony. Even my noise-canceling headphones could not handle it. My request to work more often from home or to move my desk to a quieter part of the floor was met with the response that this was not desirable because everyone needed to work in the same area to maintain team spirit. I held on; I kept up appearances, and I adapted by adding earplugs under my noise-canceling headphones and wearing tinted glasses indoors. A few months later, the first meltdowns began at work. The bathroom, the stairwell, and the archives room became my go-to places to hide “until it passed,” And after a few weeks, these crises became almost daily; I fell apart every night when I got home. I lost sleep, appetite, and weight. I spent my days utterly exhausted. I could not recover; every little sound violently jolted all my atoms. If my concentration was interrupted even once, I could not regain focus for the rest of the day. Meanwhile, the pressure on the project was mounting: gather data, publish, gather more data, and publish faster. Three research institutes shared the laboratory facilities, limiting the reservation windows. I struggled to move the project forward amid chaos and ambiguity—understand what was expected of me, make myself understood, and find quiet work time. I was drained of all substance, racing through my days at full speed without feeling much of anything. How had such an idyllic situation turned into hell?

I was already familiar with autistic burnout—not just as a concept but also as a lived experience: that collapse, not necessarily tied to a work environment, experienced by autistic individuals as a result of the overload from masking, social adaptation, and sensory stimuli. It’s not a formal diagnosis, but it aptly describes the consequences of struggling in a world that often ignores the needs of autistic people. It had previously helped me put into words those imperious vitality voids that left gaping holes in my being, draining all life and vigor from me. It would take days, sometimes weeks, to replenish those depleted reserves. During these exhaustion phases, I noticed the typical regression in skills and reduced tolerance for sounds and other sensory inputs.^1^ But what I was experiencing now surpassed anything I had previously known in terms of exhaustion, reaching new heights (or depths, in this case) of disintegration that I could never have imagined possible.

Everywhere online workplace burnout is described as a “syndrome resulting from chronic workplace stress that has not been successfully managed”^2^ with far-reaching repercussions on the brain, nervous system, cognition, physiology, musculoskeletal system, digestion, and overall health. But while this definition was helpful from a medical point of view, it seemed only to describe a flat, black-and-white reality that failed to capture the magnitude and scale of the devastating inferno I was experiencing. This information gap felt like a no-man’s land, leaving me orphaned and anonymously drowning in the throes of the conventional job market. To me, “typical” workplace burnout and autistic workplace burnout differ as much as two languages that may sound similar to the untrained ear but are built upon fundamentally different grammar. Rare studies venture into this no-man’s land, where much remains to be mapped out.^3^

To the wandering that preceded my late diagnosis of autism, with its own trail of scars, was added a new journey: the therapeutic wandering of post-burnout recovery. I did have the vital support of my general practitioner. But the health care professionals around me seemed at a loss. Even my physiotherapist didn’t know what protocol to follow when I handed him my general physician’s prescription for physical rehabilitation. No plan existed for people suffering from occupational burnout, let alone autistic individuals experiencing workplace burnout. So, I began consuming everything I could find on burnout. Having lost all ability to read and concentrate, I started with illustrations and diagrams, the simplest forms I could absorb. Slowly, I regained fragments of my reading capacity and concentration. First, I managed phrases, then paragraphs, then brief 15-minute stretches. Finally, after long and arduous months, I could read a full research article again. I read purposefully—to understand the concealed mechanisms of destruction and to piece myself back together. I knew that grasping the biopsychological mechanisms of burnout was essential to my recovery. Without a guide specific to autistic workplace burnout, I had to navigate its dimensions, dynamics, and complexities alone. This understanding was crucial for identifying the right actions and implementing proper countermeasures, giving myself the best chance to heal—and perhaps, one day, to resume my research training.

After an initial phase of decompression—during which I descended into a lethargic limbo of mental and physical exhaustion I never knew existed, mostly bedridden due to severely low blood pressure, so low that standing or even sitting upright felt impossible, engaging in minimal self-care, and unable to attempt any meaningful recovery—I gradually began to regain basic functions: bending down, moving around the house, cooking for myself, digesting food almost normally, and holding conversations lasting more than a few minutes. I also gradually recovered higher cognitive functions—such as spatial orientation, decision-making, attention, memory, and time management—through a fragmented process marked by interwoven progress and setbacks. While I could once again perform tasks like reading, concentrating, and planning, I soon realized that these functions now obeyed fundamentally different rules.

By September 2024, my mind felt sharper, quick, and focused like a laser. I am immensely relieved to have “found my brain again,” something I once feared was permanently fried. Yet, this newfound acuity coexists with a persistent inability to sustain my mental faculties for extended periods. Short-term memory impairments linger, and my energy reserves, once able to absorb stress peaks, have vanished. I navigate by sight and must carefully ration my energy, operating within narrow margins. I am progressively finding my way back to life—a life paced differently, guided by the new rhythms and limits of my embodied self.

Despite a year of Eye Movement Desensitization and Reprocessing therapy (EMDR), the flashbacks remain—less frequent and less invasive yet still sparked by the faintest sound, smell, color, or texture, drawing me back into a dark and unsettling maze of charred memories. The scar is mental, intangible, hidden among the networks of my bruised neurons. I experience four types of synesthesia, encoding memory in profoundly multimodal ways that interlace sounds, scents, images, emotions, physical sensations, and even tactile or kinesthetic impressions. This sensory affluence renders my memory remarkably infallible and stubbornly inflexible, fiercely resistant to being remodeled after the fact. Each incoming stimulus grazes the fragile edges of my recalibrated cognitive and sensory thresholds.

In the depths of my being, illuminated by the glow of lingering embers from that scorched season of burnout, the word “resilience” resonates. Is it the echo of my still-throbbing scars? Or the survival cry of my atoms? Bouncing back, springing back into shape, recovering strengths, returning to a prior state—all those punch lines fail to capture the irreversible changes I notice as I go. Resilience goes far beyond reset, adaptability, endurance, persistence, resistance, or durability; it’s an existential reconstruction—not returning to what was but learning to navigate a wholly altered reality. The post-burnout self is not a restored version of the pre-burnout self but a reassembled being with new limitations, strengths, and vulnerabilities. Beyond surviving burnout, resilience is about enduring emerging changes rather than “getting back on track,” for all traces of the previous track have been lost. Resilience is navigating fragility with grit, managing exposed synapses, reduced endurance, and the ever-present threat of relapse. It is the coexistence of paradoxes: relief and despair, sharpness and exhaustion, clarity with hypersensitivity. It is about a shattered identity and a long process of mourning the person I once was and accepting the reshaped self emerging from the wreckage. It is slow, iterative mending—reorienting and experimenting with what works. Resilience is not a goal to be achieved but a process to embrace, like learning to dance a new choreography.

In my autistic experience of workplace burnout, resilience is deeply rooted in the physical and sensory, not just the mental or emotional. It means listening with heightened attention to my body’s signals—their granularity and subtle shifts—because ignoring them now invites immediate retaliation. This includes adjusting my working hours in response to somatic cues, embracing an altered sleep rhythm, and coming to terms with the fact that I can no longer mask. It also involves recognizing that my values have shifted, likely as a way to protect me. I am, without question, irreversibly transformed.

Resilience, often symbolized by the mythical Phoenix rebirthing from ashes, is frequently oversimplified as an endless capacity to bounce back quickly from adversity. This reductive notion is harmful to everyone, autistic or not. Many narratives portray burnout as an opportunity for renewal or a fresh start. Yet, as a burnout-plagued society,^4^ it is crucial to recognize that post-burnout transformation can be so profound that “bouncing back” is not only unlikely but impossible. For autistic adults, workplace burnout often precipitates an irreversible breakdown of compensatory mechanisms and a regression in skills. Regardless of neurotype, no one emerges from burnout unscathed: its scars linger, raw and sensitive for life. While functionality may be recovered, structure is inevitably altered.

It is time to dismantle the glorification of the tired hero and the myth of endurance as the cornerstone of eternal resilience. Instead, we must implement change to prevent these irreversible alterations. We must shift from a disease-prevention to a health-promoting approach. Burnout is not a badge of honor; it is severe, grave, and dangerous, crushing lives and dreams. Let us stop romanticizing resilience and take meaningful measures so that we no longer have to invoke mythology to absolve ourselves of otherwise preventable tragedies. Autistic individuals represent an untapped reservoir of talent and creativity for society. How many more will we sacrifice before real, lasting change is made in the workplace? Neurodiversity enriches us all. It is time to honor the value of our collective potential made of neurotypical and neurodivergent individuals and create workplaces where everyone can thrive.

## Statement of Transparency

As an autistic individual with dyslexia, I utilize artificial intelligence (AI)-based assistive technology to enhance my workflow. This technology helps me overcome dyslexia-related challenges by supporting readability, translating my ideas and thoughts—which present themselves in my mind as abstract entities, bodily sensations, shapes, and feelings—into clear words, and aiding with writing and editing. It complements my skills and efforts, enabling me to navigate dyslexia-related barriers while maintaining authenticity and precision in my work. It is a tool that enhances my productivity and accessibility without replacing my unique voice or expertise. By leveraging AI in this way, I can deliver high-quality work while being transparent about the tools I use to support my neurodiverse needs.
